# When Heart Beats Differently in Depression: Review of Nonlinear Heart Rate Variability Measures

**DOI:** 10.2196/40342

**Published:** 2023-01-17

**Authors:** Milena Čukić, Danka Savić, Julia Sidorova

**Affiliations:** 1 Empa Materials Science and Technology Empa Swiss Federal Institute St Gallen Switzerland; 2 Vinča Institute for Nuclear Physics, Laboratory of Theoretical and Condensed Matter Physics 020/2 Vinca Institute University of Belgrade Belgrade Serbia; 3 Bioinformatics Platform, Hospital Clínic Barcelona Spain

**Keywords:** heart rate variability, HRV, electrocardiogram, ECG, depression, autonomous nervous system, ANS, nonlinear measures, cardiac risk, cardiovascular, mortality, heart dynamics, ECG analysis, analysis, online

## Abstract

**Background:**

Disturbed heart dynamics in depression seriously increases mortality risk. Heart rate variability (HRV) is a rich source of information for studying this dynamics. This paper is a meta-analytic review with methodological commentary of the application of nonlinear analysis of HRV and its possibility to address cardiovascular diseases in depression.

**Objective:**

This paper aimed to appeal for the introduction of cardiological screening to patients with depression, because it is still far from established practice. The other (main) objective of the paper was to show that nonlinear methods in HRV analysis give better results than standard ones.

**Methods:**

We systematically searched on the web for papers on nonlinear analyses of HRV in depression, in line with PRISMA (Preferred Reporting Items for Systematic Reviews and Meta-Analyses) 2020 framework recommendations. We scrutinized the chosen publications and performed random-effects meta-analysis, using the *esci* module in jamovi software where standardized effect sizes (ESs) are corrected to yield the proof of the practical utility of their results.

**Results:**

In all, 26 publications on the connection of nonlinear HRV measures and depression meeting our inclusion criteria were selected, examining a total of 1537 patients diagnosed with depression and 1041 healthy controls (N=2578). The overall ES (unbiased) was 1.03 (95% CI 0.703-1.35; diamond ratio 3.60). We performed 3 more meta-analytic comparisons, demonstrating the overall effectiveness of 3 groups of nonlinear analysis: detrended fluctuation analysis (overall ES 0.364, 95% CI 0.237-0.491), entropy-based measures (overall ES 1.05, 95% CI 0.572-1.52), and all other nonlinear measures (overall ES 0.702, 95% CI 0.422-0.982). The effectiveness of the applied methods of electrocardiogram analysis was compared and discussed in the light of detection and prevention of depression-related cardiovascular risk.

**Conclusions:**

We compared the ESs of nonlinear and conventional time and spectral methods (found in the literature) and demonstrated that those of the former are larger, which recommends their use for the early screening of cardiovascular abnormalities in patients with depression to prevent possible deleterious events.

## Introduction

Cardiovascular diseases (CVDs) are the number one cause of death globally according to the World Health Organization [1,2]. Depression is the number one mental health–related contributors to the global burden of disease [3]. When combined, these 2 diseases can lead to increased mortality risk [4-6]. Recently, the European Society of Cardiology published a position paper about the mechanisms linking depression and CVD, based on abundant evidence from literature [7]. Although this connection was discovered a long time ago [8-10], the CVD screening of patients with depression is still far from routine.

In nearly 70% of patients with depression, somatic symptoms, such as lack of energy, sleep disturbance, lack of appetite, decreased sex drive, general pains, etc, dominate the clinical picture [11]. These symptoms are due to autonomous nervous system (ANS) dysfunction. Heart rate variability (HRV) is regulated by the ANS, and its disturbance is a marker of CVD. The relation between HRV and depression has been well understood [7,12-15]. We registered at least 14 reviews that meta-analytically compared conventional methods of analysis of this relation [16-28].

Medical professionals interested in the detection of depression may be uninformed of the knowledge and methods offering additional insights into a patient’s condition, with the knowledge coming from theoretical research—mathematical analysis, complex systems dynamic theory, and information theory. These methods can be used to extract information embedded in electrophysiological signals, represented as time series—electrocardiogram (ECG), electroencephalogram, electromyogram, etc. Current view of what electrophysiological signals can yield is quite obsolete and limited by a reductionist approach established in clinical practice, because most devices for recording physiological signals have built-in algorithms based on Fourier analysis [29]. These standard (time and frequency) methods of electrical signal analysis are designed for (predictable) electro-mechanical systems and are not well suited for (complex) physiological systems. A number of review studies [16-27] offer very detailed comparative analyses of time and frequency measures of HRV related to depression. They rely on the assumption that the dynamics of the system may be linearized, where valuable information is lost in the case of electrophysiological signals.

Physiological systems are complex. Complex systems are composed of multiple subunits that interact in a nonlinear fashion producing unpredictable behaviors [29,30]. Although homeostasis is usually perceived as a still condition, “healthy heartbeat displays highly complex, apparently unpredictable fluctuations even under steady-state conditions” [29], whereas heart failure, for example, shows “slow periodic oscillations that correlate with Cheyne-Stokes breathing” [31]. The theory of complex dynamic systems applies to such a system. Its behavior can be predicted at best for short intervals, and it is characterized by long-range correlations and organized variability.

In information theory, the rate at which a system is producing information is described by Shannon entropy (ShanEn)—a quantity reflecting the number of possible states a system can occur in, that is, the level of uncertainty (unpredictability). Pincus et al [32,33] adapted the ShanEn for cardiology research and devised the approximate entropy (ApEn) algorithm, a statistic quantifying serial irregularity. Further, Richman and Moorman [34] refined this measure into sample entropy (SampEn), which was later improved by Costa et al [35], proposing the multiscale entropy (MSE) algorithm that calculates irregularity changes on multiple scales [35]. Costa et al [36] performed a series of studies focusing on methods of analysis of ECG, and their work was a significant step in the acceptance of nonlinear methodology. Translated to signals, the higher the entropy, the higher the irregularity of a signal, which is most often interpreted as higher complexity. This “awkward fact,” as Vargas et al [30] noted, is paradoxical as complexity assumes a structure that is highly ordered. Glass and Mackey [37] stated that “Random outputs result from degraded control mechanisms and/or breakdown of the coupling among them,” that is, from the loss of complexity. Nevertheless, as much as this confusion makes the insights into control mechanisms more difficult, the measures of complexity or irregularity differ between health and disease rendering them suitable for nonspecific markers of ill-health.

Neural control mechanisms, which demonstrate fractal properties, generate “organized variability” (previously thought to be the “noise” in the signal) characteristic of a healthy physiological system [38]. Physiological systems are scale-free; self-similar fluctuations are observed on different time scales. From one moment to the next, the fluctuations detected in the same signal are quite variable [31,32]. A system that is fractal can demonstrate irregularity across a wide range of scales, but the type of “disorder” or “roughness” on different scales is statistically similar [39-41]. Goldberger et al [31] stated that “organized variability is an inevitable consequence of fractal self-organization.” According to the number of publications (in cardiology), the most popular fractal-based methods in use for analyzing HRV is detrended fluctuation analysis (DFA), which is based on correlation properties and uses random walk [42].

In interpreting the results and choosing the measures to be used, the physical meaning of the applied nonlinear analysis and the physiological context of a particular disease have to be kept in mind. Beside entropy- and fractal-based measures, there are other families of nonlinear measures that are methodologically very different. Poincaré plots are among the most accurate measures applied in cardiology [43], and being a graphical representation, they are very convenient for clinical application. Largest Lyapunov exponents (LLE) [44], which were used often in the beginning of the field, detect the level of chaos in a signal. Lempel-Ziv complexity (coming from information theory) quantifies the uncertainty contained in time-series data and is still among the frequently used measures in physiology [45]. Several correlation-based measures also showed promising results, but to describe even the basic methodology for all of them is out of the scope of this manuscript.

The fundamental difference between irregularity statistics and conventional variability measures is that the conventional approach is focusing on tasks of quantifying the degree of spread around the central value while the order of the input data is irrelevant; whereas in irregularity statistics, nonlinear measures track changes from random to very regular and the order of samples is essential to the algorithm [40]. Nonlinear measures have been shown to be very effective in detecting the slightest differences between healthy and ill heart dynamics—even when time series of the compared states are varying around almost the same mean values [29,41]. An impressive example of the advantage of nonlinear methods is the case of detecting sudden infant death syndrome based on entropy measures calculated from ECG; the standard method was not able to detect any difference between healthy and babies under a serious risk [41].

This study is a random-effects meta-analysis of the most important studies that used nonlinear methods to confirm the connection between HRV (as a marker of CVD) and depression. We calculated effect sizes (ESs) from these studies and compared them with the ESs of standard (conventional) methods found in the literature. The aim of this work was to help convince clinicians to (1) introduce cardiological screening to patients with depression, since depression is confirmed to bear a risk for CVD [10,16,22,46-48] and (2) apply nonlinear methods to HRV analysis for more accurate and reliable screening results.

## Methods

### Overview

Since there are a considerable number of recently published meta-analytic studies [16-27] regarding the classical (spectral or conventional) approach to analyzing HRV, we decided to include only those studies that performed any nonlinear method of analysis or had mixed analytic approaches (applying both standard and nonlinear methods of analysis of heart rhythm to compare the effectiveness of analytics). This meta-analysis was performed in agreement with PRISMA (Preferred Reporting Items for Systematic Reviews and Meta-Analysis) guidelines [49] with the main aim to present an integrated, realistic ES of the nonlinear measures in distinguishing between major depressive disorder (MDD) and controls, in comparison to conventionally used measures reported in previously published meta-analytic studies. Our work does not compare effects of interventions; thus, it was not preregistered (ie, the review protocol registration does not exist).

Only case-control studies or longitudinal studies that used ECG recordings or measurements of heart rate or any automated ECG diagnosis (for example, including early arrhythmia detection) and successive nonlinear analyses were included in our pool. Our query was kept as broad as possible to retrieve as many relevant papers as possible. We searched web resources, such as Springer, Wiley, Elsevier, IEEE, National Institute of Mental Health, Frontiers, PlosOne, Hindawi, Web of Science, PubMed, Cochrane Library, Scopus, and National Center for Biotechnology Information, with sets of keywords: (“depression” OR “Major depressive disorder” OR “bipolar depression”) AND (“ECG” OR “electrocardiography” OR “HRV” OR “heart rate variability”) AND (“Nonlinear analysis” OR “Fractal analysis” OR “entropy”).

We then scrutinized the abstracts and full texts (in English) and discarded those that were purely theoretical considerations of the connection of HRV and depression without the quantification of HRV measures (even when they included keywords from all 3 sets); papers on mood disorders without separate data on depression; studies where samples comprised solely of healthy subjects or subjects under the age of 18 years; studies without age-matched controls; animal studies; and papers without peer review.

Depression was diagnosed using the Diagnostic and Statistical Manual of Mental Disorders (DSM; DSM-III-R, DSM-IV-TR, and DSM-5), International Classification of Diseases 10th Revision, Mini-International Neuropsychiatric Interview, Beck Depression Inventory, or Montgomery-Åsberg Depression Rating Scale. Some studies, found as references through hand searching of citation lists in review papers, were downloaded from the ResearchGate platform or were included even if nonretrievable as full text. Papers with standard analyses of ECG, added for comparison, were found in part via web-based search, from reference lists of review papers, and from the authors who were kind to send us full texts. The web search was performed in February and March 2021. Our screening was finalized in October 2021.

During the process (after removing the duplicates), we kept the working sheet (updated by all authors) with the basic data extracted from every paper included in the study (the first author’s name and the year of publication as an identifier, sample size, the mean and SD calculated for groups, the measures used in the research, the effect detected, and specific observations about the accuracies of applied analyses). Where we were unable to extract the data, we used WebPlotDigitizer software [50]. After coding all the data from the included papers, ES estimates were transformed into the same metric to be compared (Cohen’s *d_s_* was corrected, hence having the same value as Hedges’ *g*). We used the *esci* module in jamovi exploratory software (open-source statistical software written in R) [50] to calculate overall ESs (correction of Cohen’s *d*) and 95% CIs and to generate forest plots [51]. The forest plots were used to visually display the individual and overall ESs.

ES is a quantitative description of the strength of evidence about a phenomenon. Cohen’s *d* describes the standardized mean difference of an effect [52-55]. In between 2 groups of independent observations, Cohen’s *d_s_* is:



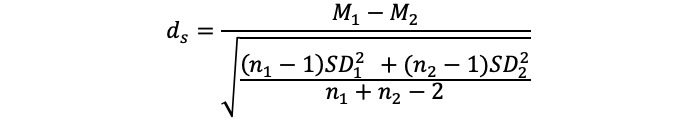



where *M_1_* and *M_2_* are the variable means of the 2 groups (patients and controls); in the denominator, the pooled SD is the Bessel correction for bias in the estimation of population variance (based on the least squares estimator [54,55]); *SD_1_* and *SD_2_* are the respective SDs; and *n_1_* and *n_2_* are the sample sizes of the groups. Cohen’s *d_s_* is also directly related to the *t* test:



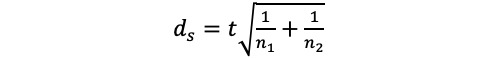



where *t* is the *t* statistic, and *n_1_* and *n_2_* are as above. This is a direct relation between the ES and statistical significance. Here, statistical significance is expressed regardless of whether the 95% CI around Cohen’s *d_s_* includes 0 or not. Hedges and Olkin [54] showed that the formula for Cohen’s *d* based on sample averages gives a biased estimate of the population ES (especially for smaller sample sizes, n<20). The Cohen’s *d* that we calculated is actually Cohen’s *d_s_*, described in Lakens [55] (where SDs are pooled as in the formula above, not a single average of both SDs from samples 1 and 2). Thus, it is the Cohen’s *d* of a sample, *d_s_*. Further to be corrected for biases, according to Hedges and Olkin [54], it must be multiplied by another Bessel correction (1 – 3 / 4(N1 + N2) – 9) [55]. After calculating the corrected Cohen’s *d_s_* for all the included studies, by applying pooled SD in the process, we confirmed that what is calculated as a correction (for biases) in the *esci* module in jamovi software is actually Hedges’ *g*. The authors of the software also describe that the product of their calculation (included in forest plot that the program is generating) is equal to Hedges’ *g* [51,56]. In several studies that reported *t* values, we calculated Cohen’s *d_s_* according to the second formula. This interpretation was done based on previous literature [51-56].

### Ethical Considerations

Since all the studies included in our review have already received prior approvals from their local ethics committees, and we did not use nor collect any additional data from the patients and only reanalyzed already published data, we do not report any ethic approval for this particular study.

## Results

**Figure 1:**
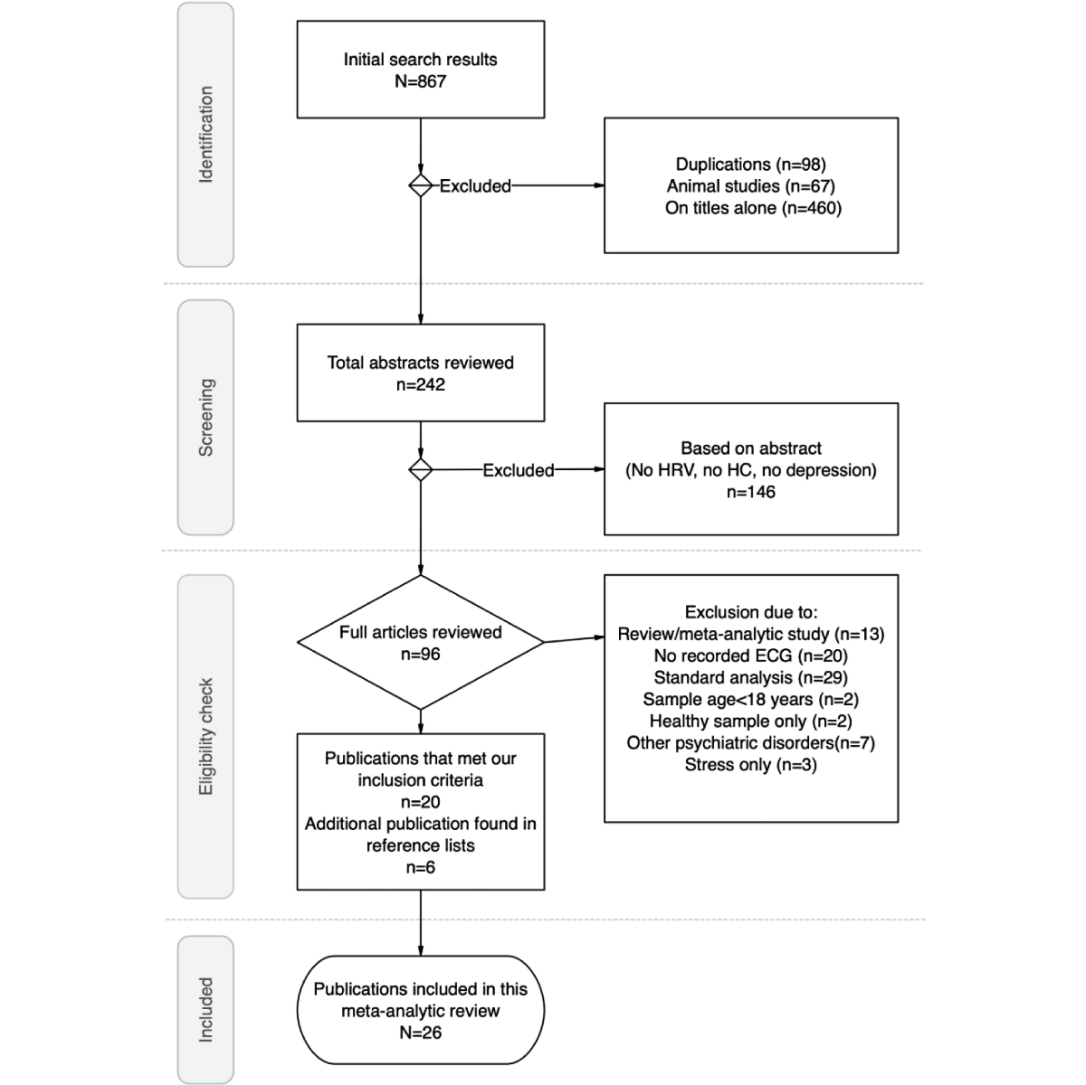
Flow chart representing the procedure of choosing 26 studies included for this review. ECG: electrocardiogram; HC: healthy controls; HRV: heart rate variability.

Our initial search (based on the logical formula given in *Methods*) in the abovementioned web services yielded 867 papers. The elimination was performed through phases shown in the flow chart (Figure 1) showing the identification, screening, eligibility check, and inclusion of studies in accordance with PRISMA 2020 [49]. The chosen 26 papers originated from the following databases: Elsevier (n=10), PubMed (n=8), Frontiers (n=2), Web of Science (n=4), Springer (n=1), and IEEE (n=1). They encompassed a total of 1537 patients diagnosed with depression and 1041 healthy controls. The studies included those that used nonlinear analyses or both nonlinear and standard analyses.

Direct quantitative comparisons could not have been made, as studies varied in methodologies, as well as in research questions—detecting biomarkers or predictors of depression, CVD mortality risk estimation or risk analysis, effects of different therapies, etc. Therapies included those exploring medication effects or spillover on HRV and psychological or psychiatric interventions; some examined inflammation or other important physiological markers, but some also used historical medical data (for example, from Medicare archives in the United States, see [57-59]). As the effects of therapies are not the topic of this paper, we compared the studies grouped by family of measures used (in nonlinear analysis) and summarized their results and conclusions concerning only the detection of the relation between depression and CVD mortality risk.

**Figure 2:**
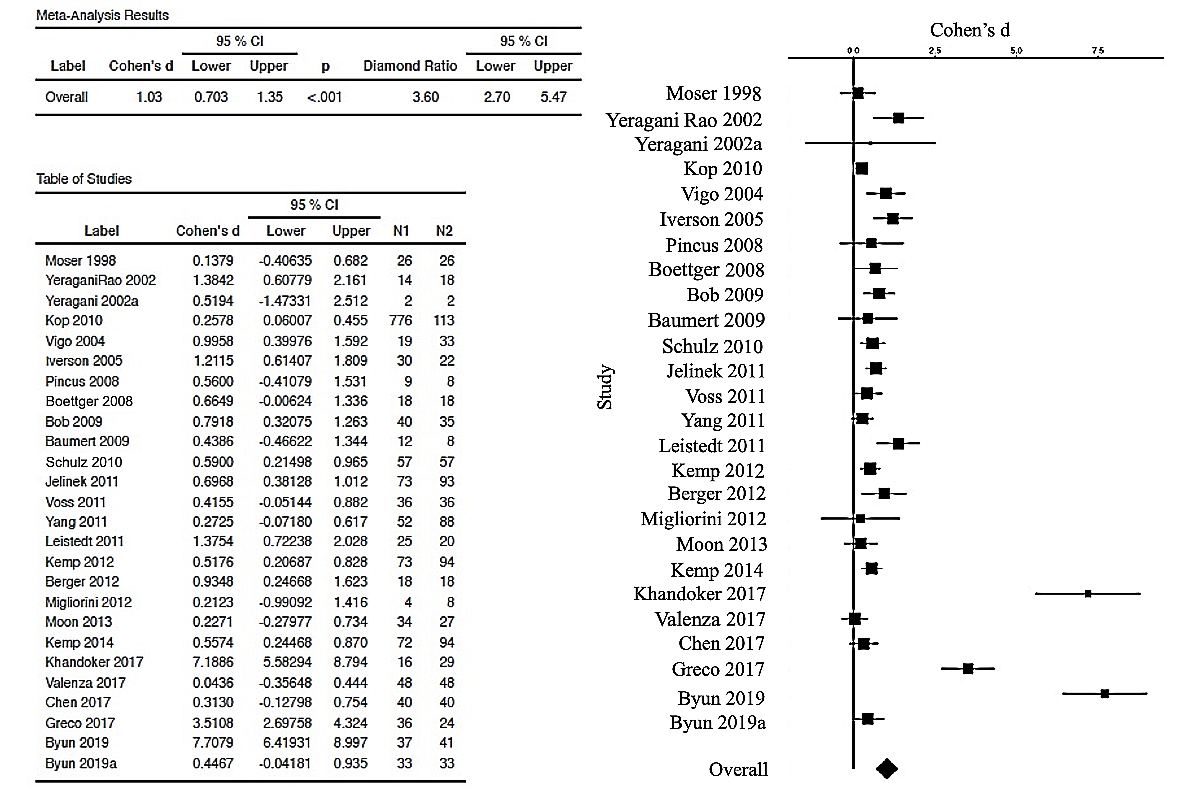
Forest plot and the table of random-effects meta-analysis showing the corrected effect size and CIs, as well as both sample sizes (N1: patients diagnosed with depression; N2: controls). For each study performing more than one method of nonlinear HRV analysis, we presented the largest effect size. Both table and forest plot are generated by the esci module in jamovi software. In all, 15 (58%) out of 26 included studies were shown to have statistically significant results. The overall effect size (unbiased/corrected) is 1.03 (diamond ratio 3.60). HRV: heart rate variability. [14,44,45,57-79].

Figure 2 shows the information about the studies’ methodologies (the majority [19/26, 73%] used more than one; only 7 [27%] studies were based on one nonlinear measure) and their conclusions. After initial random-effects meta-analysis of overall ESs of all included studies, we identified 3 distinct (methodological) groups of research and performed 3 additional meta-analyses. The first group used DFA (8 studies) with reported Cohen’s *d_s_* (corrected for biases) and 95% CIs. The second group of studies used methods from the large family of entropy measures: ApEn in 5 studies, SampEn in 5 studies, MSE in 3 studies, and ShanEn, Renyi entropy and refined composite multiscale entropy, and multilag tone-entropy, each in one study. The third group comprised various nonlinear analyses: Poincaré plots (n=4), LLE (n=2), symbolic measures (n=2), Lempel-Ziv complexity (n=1), complex variability, mutual information, autonomic information flow, beat decay NN, logarithmic respiratory sinus arrhythmia, recurrence plot analysis, Complex Correlation Measure, correlation dimension, and Katz fractal dimension. This group also demonstrates a historical order in which nonlinear measures entered cardiology, and some of them are still very popular in health applications (for example, LLE or Poincaré plots). Besides, this “historical” group demonstrated an average ES more than 2 times higher than any prior conventional approach, to the best of our knowledge.

**Figure 3:**
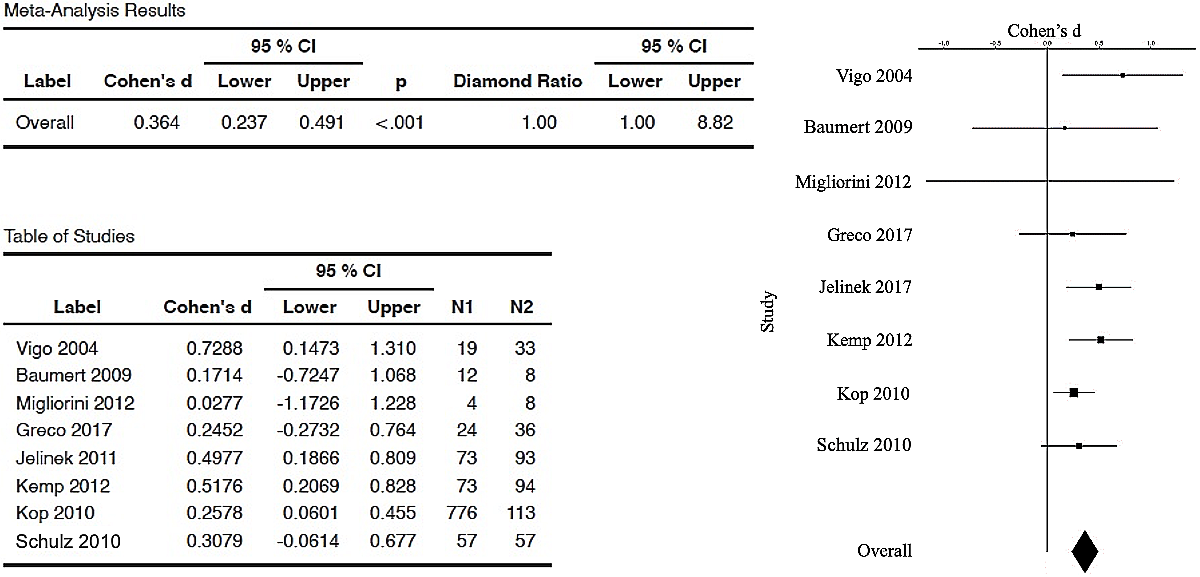
Forest plot (and table) showing the random-effects meta-analysis of a group of selected papers (8 publications [45,58,59,64,73,74,77,79]) that used detrended fluctuation analysis generated by the esci module in jamovi software. In all, 4 out of 8 studies were shown to have statistically nonsignificant results, with the overall effect size (biases corrected) being 0.364 (between small and medium effect, closer to medium, according to Cohen [52]), and the diamond ratio is 1.

**Figure 4:**
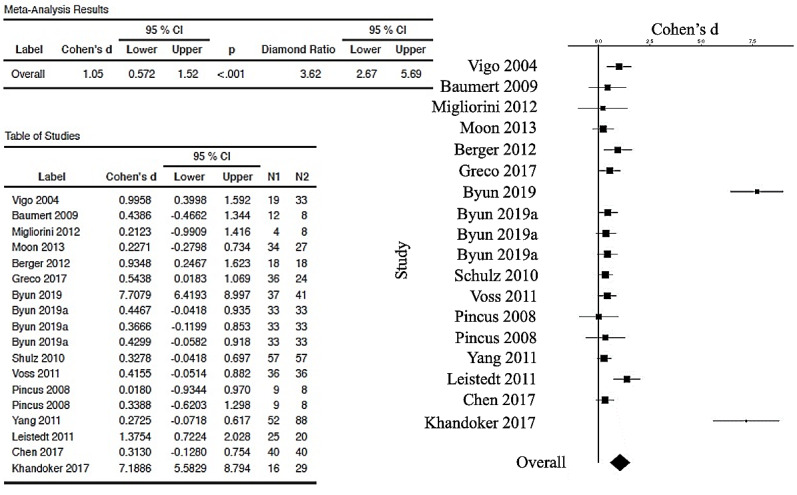
Forest plot (and table) showing the random-effects meta-analysis of a group of selected papers (15 publications [45,57,60,63-67,69,70,72,73,77-79]) that used entropy measures: approximate entropy (ApEn), logarithmic ApEn, sample entropy, fuzzy entropy, Shannon entropy, cross entropy, Renyi entropy, multiscale entropy, and improved refined composite multiscale entropy, generated by the esci module in jamovi software. The overall effect size for this group is 1.05 (which is very large, according to Cohen [52]). In all, 10 out of 18 studies were shown to have statistically nonsignificant results. Interestingly, Byun et al [60] calculated 4 entropy measures and demonstrated that only Shannon entropy yielded a highly useful effect size of 7.7 (which is the best result detected in this entire pool of publications, followed by Khandoker et al [72] with a corrected effect size of 7.3).

**Figure 5:**
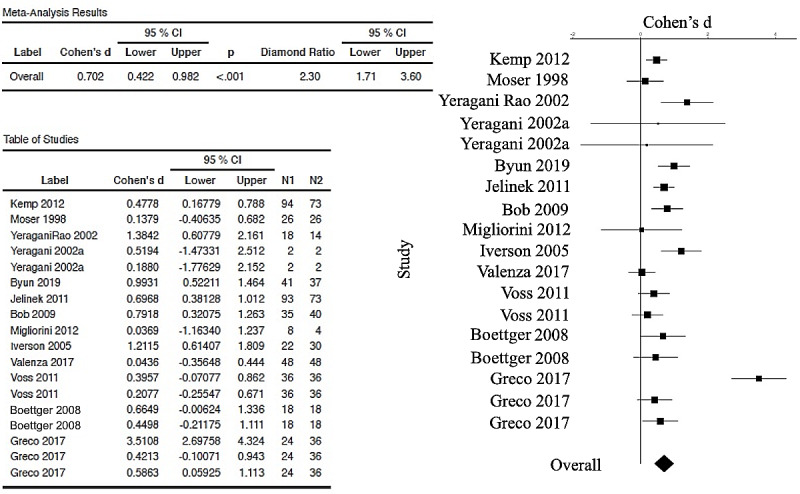
Forest plot (and table) showing the random-effects meta-analysis of group of selected papers that used a number of different nonlinear analyses of HRV in depression, excluding entropy and detrended fluctuation analysis measures. Those are Poincaré plots (SD1 and SD2), largest Lyapunov exponents, symbolic measures, Katz fractal dimension, correlation dimension, Complex Correlated Measure, mutual information, logarithmic respiratory sinus arrhythmia, heart rate turbulence, Lempel-Ziv complexity, recurrence plot analysis (determinism and recurrent rate are the most prominent result in Greco et al [73], ds=3.5), autonomic information flow, and beat decay NN. The plot and table are generated by the esci module in jamovi software. The overall effect size of studies included in this group is 0.7 (diamond ratio 2.3), which is considered large. Eight studies from this group were shown to have practically meaningful results: Kemp et al [74], Yeragani, Rao et al [44], Byun et al [70], Iverson et al [75], Jelinek et al [59], Bob et al [76], and Greco et al [73] (2 out of the 3 applied measures included and represented in this group). [44,45,59,61,62,65,68,70,71,73-76].

The forest plot (and the table with Cohen’s *d_s_* corrected and 95% CIs) is used to visualize those meta-analytic comparisons of the ESs. Figure 2 represents the overall meta-analytic comparison of the best ESs of the 26 included studies. Figure 3 represents meta-analysis of the DFA group (with 8 studies compared, with an overall ES of 0.364, 95% CI 0.237-0.491). Figure 4 represents meta-analysis of the entropy group (15 studies compared, with an overall strong ES of 1.05, 95% CI 0.572-1.52). Figure 5 represents all other nonlinear methods used in the examined studies (13 studies compared, with an overall ES of 0.702, 95% CI 0.422-0.982). All mentioned comparisons yielded *P*<.01 (as shown the tables in all the figures). As the majority of studies applied several types of nonlinear analysis, we did multiple comparisons of corrected ESs that we separately calculated for each method. The best ES (*d_s_*=7.7, 95% CI 6.4193-8.997) was obtained for the study [60] that used 4 entropy algorithms (ApEn, SampEn, ShanEn, and fuzzy entropy). When calculating ESs for each entropy method separately, ShanEn performed the best, and fuzzy entropy did not yield a significant result.

From overall meta-analytic comparison of ESs, out of 26 studies included, 11 were shown to be statistically insignificant [59-70], and one paper [71] had the lower CI touching the zero line, implying border significance. Thus, 15 (58%) out of 26 studies were statistically significant, with the overall ES of corrected Cohen’s *d_s_*=1.03 (elsewhere also reported as Hedges’ *g*), which is a large ES [52] and can be translated to more than 1 SD [55].

## Discussion

Our results show that the overall standardized ES of nonlinear measures of HRV in depression overperforms the ESs of conventional measures of HRV reported in the literature. Although the overall ES in our comparison (all 26 studies) was 1.03, the best entropy-based group ES was 1.05, the DFA group ES was 0.36, and the third, miscellaneous group yielded an ES of 0.70. In the latest standard HRV measures-based study [28] (which is very similar to ours by the number of included studies), the ESs of several conventional measures varied up to 0.46. The meta-analysis by Rottenberg [13] reports a small ES (*d*~0.2), which explains only about 2% of the overall variance for conventional measures. Many of the published papers reported nonsignificant and mild-to-moderate ESs, whereas we found much larger effects: for example, the best ES in our research reached the value of 7.7 [60], and several others demonstrated higher ESs than those reported in conventional analyses (eg, the ES in Khandoker et al [72] was 7.3, which is very large according to Cohen [52]; in Greco et al [73], the ES was 3.5; and several others reached an ES around 1).

As commented in *Results*, almost half of the included studies did not have a significant effect in discriminating patients with depression from controls. This might be because not all patients with depression have disturbed heart function and because of the modest sample sizes. Indeed, the majority of those authors concluded that their initial results were promising but required replication. Later studies (2010 onward) show that researchers started using larger data sets or at least existing databases [58]. In the last 10 years, studies not only started using nonlinear measures but also combining them with some forms of machine learning to discern MDD [60,72,73,77,81,82]. We consider this methodological combination promising, especially since our previous work based on depression detection from electroencephalogram yielded good results [83-86].

Through the list of nonlinear methods presented in *Results*, we can follow the evolution of the understanding of how to interpret the results of nonlinear analysis. First, it was LLE, but since Lyapunov exponents mainly serve to detect chaos in signals, they can hardly be used for the precise delineation of groups. Then, in several papers, symbolic methods were used and reported as being successful, but again, their interpretation was problematic for which they were practically abandoned. The most promising family of nonlinear measures is the one based on entropy, in particular, ShanEn, then SampEn and ApEn (maybe also logarithmic ApEn); MSE seems a little more difficult to interpret. Irregular signals have higher entropies. Increased irregularity can point to a degradation of internal control mechanisms, or as Goldberger [29] puts it, decomplexification that is characteristic of aging and disease. Additionally, DFA, as a fractal method (as well as several other methods of calculating fractal dimension and correlation dimension) makes sense, since neural control mechanisms are shown to have a fractal nature. In fact, all spectral measures calculated from ECG are a function of RR intervals length and are correlated; they do decrease with aging, but in disease that change is much more pronounced, and the function is lost [87-89]. In that sense, DFA is accurately detecting short- and long-term correlation that are important for healthy heart dynamics but also its synchronization with breathing [82,90]. Among other measures, the Complex Correlated Measure applied by Jelinek et al [59] performed quite well. Some recurrence plot analyses (Poincaré plot analysis and generalized Poincaré analysis) that quantify self-similarity in the processes were also used with good results [91]. In the reviewed literature, there are also combinations and alterations of the mentioned methods of analysis, such as combining the Poincaré analysis with DFA, applying Pearson coefficients on prior Poincaré analysis, or choosing the most prominent coefficients from several analyses and combining them as successful features for classification.

Nonlinear HRV analysis might be used as an aid in differential diagnosing or in indicating comorbidities [14,20,43,92]. For example, Chang et al [82] succeeded in distinguishing between bipolar II depression and unipolar depression, based on SampEn analysis of the HRV of 707 subjects. Kemp et al [14] found that anxiety disorders comorbid to MDD, most of all generalized anxiety disorder, contributed to the reduction of HRV. They elaborated on how nonvagal components of heart rate might further distinguish between subtypes of the disorder [20,43,92].

Cardiac vagal control (CVC) is associated with both physical and mental health. Low CVC is considered to be an indicator of risk of cardiac disease, including myocardial infarction and congestive heart failure [10,93]. Since variability in heart rate that is gated by the respiratory cycle [13,43] reflects the extent of CVC, it is logical to analyze its nonlinear dynamics and its aberrations to detect and treat depression. It could be a link between the polyvagal theory of Porges [94] and the physiological complexity (decomplexification and stereotypy of disease) of Goldberger [95]. In parallel to the polyvagal theory, there is also the neurovisceral integration model [96], both emphasizing the importance of taking into account ANS aberrations, along with the existing need to improve psychiatric nosology.

Important insights about healthy heart dynamics and how it changes with aging and disease were published in the 90s and served well the detection of several pathological entities [41,90,97]. We have learned that the mechanisms of neural control are fractal in nature (scale-free) and that they generate the so-called complex variability (once believed to be a background noise to the signal), which is a characteristic of healthy heart dynamics. In pathological states, one can observe a characteristic loss of complexity (decomplexification) that leads to recognizable oscillatory (predictable) behavior of a complex system, reduced to a single scale or frequency. The aberrated dynamics can be precisely quantified by fractal and nonlinear measures. The standard idea of comparison of healthy and ill organism pertains to the calculation of traditional mean values, SDs and the like, from electrophysiological signal (here ECG). When one compares the recording of a healthy heart with one of a patient diagnosed with congestive heart failure, their calculated means are within the same SD. However, it can be seen even with a naked eye that those signals are different (in dynamics and structure). Traditional methods do not show a significant difference here. The stereotypy of disease, as Goldberger explained [31], is connected with the decomplexification of a dynamical system’s output, observed in early complexity studies. Complexity analysis can complement this clinical heuristic with adequate mathematical tools to quantify the changes in a patient’s state.

Too aggressive preprocessing of the data can contribute to misleading results due to the loss of the exact order of samples. The history of the system is important in knowing its dynamics: earlier samples—the values of a physical phenomenon we measure (here in microvolts)—affect the later values, and if you shuffle the order, you lose the internal nonlinear structure that is contained in the sequence of those samples; this is called the historicity of data. Thus, it is necessary to analyze raw sequences of the records (broadband signal is the most information rich). This might be the reason why the nonlinear methods are superior to conventional ones.

The data can be easily obtained by novel portable ECG monitoring devices that are approved as medical-grade signal quality equivalent to a Holter monitor but are much more practical and comfortable to use by the patient herself or himself, taking only a couple of minutes. The data can then be processed by a combination of nonlinear analytics and advanced statistical procedures (to control, for example, for comorbidities and other confounding factors or for feature selection for further machine learning). Even better, the analysis can be empowered by machine learning applications that are widely in use due to the high power of computation and cloud computing [83-86,97].

To conclude, the ESs of nonlinear methods are larger than those of standard methods in HRV analysis. Measuring ECG and applying nonlinear analysis of HRV should enter the routine clinical practice for patients with depression. Although Porges [94] states that psychiatrists and psychologists seem not to be sufficiently interested in the use of objective biomarkers in their daily diagnostic work with patients with depression, the real question here is how ethical it is to keep this status quo and apply trial and error protocols in depression treatment without prior objective screening for CVD risks.

## Abbreviations

ApEn: approximate entropy
ANS: autonomous nervous system
CVC: cardiac vagal control
CVDs: cardiovascular diseases
DFA: detrended fluctuation analysis
DSM: Diagnostic and Statistical Manual of Mental Disorders
ECG: electrocardiogram
ES: effect size
HRV: heart rate variability
LLE: largest Lyapunov exponents
MDD: major depressive disorder
MSE: multiscale entropy
PRISMA: Preferred Reporting Items for Systematic Reviews and Meta-Analysis
SampEn: sample entropy
ShanEn: Shannon entropy
